# Comparison of Clinicopathological Characteristics for HER2-Null, HER2-Ultralow and HER2-Low Breast Cancer: A Single-Center Study

**DOI:** 10.3390/medicina61040719

**Published:** 2025-04-13

**Authors:** Seval Akay, Mumin Emiroglu, Canan Kelten Talu, Olcun Umit Unal

**Affiliations:** 1 Department of Medical Oncology, Izmir City Hospital, Izmir 35540, Turkey; 2 Department of Pathology, Izmir Tepecik Education and Research Hospital, Izmir 35020, Turkey; muminemiroglu93@gmail.com; 3 Department of Pathology, UHS Izmir Faculty of Medicine, Izmir Tepecik Education and Research Hospital, Izmir 35020, Turkey; esracanankelten.talu@sbu.edu.tr; 4 Department of Medical Oncology, UHS Faculty of Medicine, Izmir City Hospital, Izmir 35540, Turkey; drolcun@hotmail.com

**Keywords:** ADC, breast cancer, HER2-low, HER2-negative, HER2-null, HER2-ultralow

## Abstract

*Background and Objectives*: A recent clinical trial has demonstrated that breast cancer with low-HER2 expression levels responds to trastuzumab deruxtecan treatment. This has prompted a re-evaluation of HER2-targeted therapies in the HER2-negative group. Further research is required in the form of more detailed information about HER2-negative breast cancers with HER2-null, HER2-ultralow, and HER2-low subgroups. This study represents a novel approach to this field. *Materials and Methods*: HER2-negative breast cancer patients were classified into three groups as HER2-null, HER2-ultralow, and HER2-low. A comparison of clinicopathological features was analyzed retrospectively. *Results*: Of 722 patients, 22.3% were HER2-null, 23.7% were HER2-ultralow, 54.0% were HER2-low. While two-thirds of all the patients were evaluated as having T2 tumors, T4 tumors constituted 2.4%. Among HER2-negative cases, 11.8% were triple-negative and 88.2% were hormone-positive. The mean tumor diameter was 0.57 cm larger in the HER2-ultralow group than in the HER2-null group and 0.34 cm larger in the HER2-low group than in the HER2-null group. HER2-null tumors tend to be smaller. The HER2-low group was more likely to relapse than the HER2-null group. There were no significant differences in the distribution of hormone positivity or negativity (TNBC) among the groups; they accounted for 89.2% and 10.8% of all cases, respectively. *Conclusions*: HER2-negative breast cancer is a heterogeneous disease and deserves a detailed review in terms of diagnosis and treatment. HER2-ultralow tumors are larger in size and have a prognosis comparable to HER2-null tumors. HER2-low tumors tend to recur much more frequently and with poorer outcomes. In this field, new therapeutic approaches may result in better outcomes.

## 1. Introduction

Breast cancers are commonly classified based on the presence or absence of certain receptors, including estrogen receptors (ERs), progesterone receptors (PRs), and human epithelial receptor-2 (HER2). HER2, also known as ERBB2, is a gene that encodes a protein involved in cell growth and division. In normal cells, HER2 helps to regulate cell growth, but when HER2 is overexpressed or amplified, it can lead to uncontrolled cell growth and cancer.

HER2-positive breast cancers accounts 15% of all breast cancer cases [1]. Immunohistochemistry (IHC) or in situ hybridization (ISH) is employed to assess the status of the HER2 receptor. For HER2-positive breast cancers, HER2-targeted therapies led to better survival, which are historically known as having poor prognostic ability [2]. Improved standards of care and new therapeutic options in HER-2-positive breast cancer management have reversed this unfavorable situation [3].

So far, HER2-targeted therapies have been restricted to use within the HER2-positive group of breast cancer patients [4]. Recent data show efficacy in patients with so-called HER2-negative breast cancer, although a new generation of HER2-targeted therapies has prompted interest in identifying which patients in this group are most likely to benefit from these therapies. While HER2-negative breast cancer typically denotes tumors that lack HER2 overexpression, this statement has recently been slightly expanded, and it has been reclassified into three new subgroups: HER2-null, HER2-ultralow, and HER2-low. Approximately 50% of all cases of breast cancer are HER2-low. This category of tumor has been demonstrated to respond effectively to treatment with trastuzumab deruxtecan (T-Dx) [5]. Concurrently, investigations into the efficacy of the same treatment in HER2-ultralow tumors are underway.

The objective of this study was to identify the differences and similarities between breast cancer patients with HER2-null, HER2-ultralow, and HER2-low expression in the Turkish population and to determine their clinicopathological characteristics and prognosis.

## 2. Materials and Methods

This is a single-center and retrospective analysis conducted at Izmir Tepecik Education and Research Hospital. Patients diagnosed with HER2-negative breast cancer between 2011 and 2023 were included in the study. The diagnosis was established based on tru-cut biopsy specimens or surgical materials. Data were retrieved from the hospital’s electronic database, comprising a total of 722 patients who met the inclusion criteria.

The study population consisted of patients aged 18 years or older who had a confirmed diagnosis of HER2-negative breast cancer. Cases with available clinical and pathological data were included. Additionally, consultation specimens were also considered eligible, provided that sufficient clinical information was accessible, even if the original tumor size was missing.

Patients were excluded from the study if they had bilateral breast tumors or had received chemotherapy within the last five years for any malignancy. Furthermore, cases with insufficient clinical data, particularly those with biopsy results but without further clinical information, were not included.

All pathological evaluations, including initial diagnosis and potential re-evaluations, were conducted by a single expert pathologist who is an expert in breast cancer. This ensured consistency in diagnostic criteria and pathological assessment.

Histopathological evaluation of ER and PR is based on the percentage of nuclear staining (the ratio of positive tumor cells to all tumor cells) and nuclear staining intensity (the intensity of staining in positive tumor cells). Cytoplasmic staining or <1% nuclear staining of cells is considered negative. Conversely, ≥1% nuclear staining is considered positive. Tumors expressing ER > %1 or PR > %1 are defined as hormone-positive tumors. HER2-negative tumors that showed ER > 1% or PR > 20% are categorized as luminal tumors, those with Ki67 < 14% are called Luminal A, and those with Ki67 > 14% are called luminal B. The hormone-negative group was defined as triple-negative breast cancer (TNBC) regardless of Ki67 status. Tumors that were ER-negative but had PR < 20% were also included in the TNBC group.

The patients were classified according to their level of HER2 expression as follows: HER2-null, HER2-ultralow, and HER2-low. The classification of these HER2 definitions is based on the staining of tumor cells with immunochemistry dyes (Table 1).

The patients were stratified according to HER2 status considering age, menopausal status, tumor site (right/left), tumor number (single/multiple), tumor cell type (ductal/lobulary/others), tumor nuclear and histological grades, the absence or presence of necrosis and DCIS, ER, PR, and Ki67 levels, HER2 status, luminal types, tumor size and T stage, number of lymph nodes involved and N stage, disease stage, absence or presence of local recurrence, relapse and metastasis, surgical procedure, treatment selection, metastasis region (if present), and survival.

The SPSS 25.0 program (IBM, Armonk, NY, USA) was used for statistical analyses. Descriptive statics, including the mean, median, and standard deviation, were used to summarize continuous variables, while categorical variables were presented as frequencies and percentages. The Chi-square test was employed for comparative analyses of the HER2-null, ultralow, and low groups, while independent *t*-tests or Mann–Whitney U tests were applied for continuous variables, depending on normality assumptions. The Kolmogorov–Smirnov test was employed to ascertain whether the data fit a normal distribution, while results below this value were not considered to be in accordance with a normal distribution. The Kruskal–Wallis test was employed for the analysis of non-normally distributed groups, while Tamhane’s T2 test was utilized for the analysis of subgroups. The one-way ANOVA test utilized to compare multiple variations.

A Cox regression model was utilized to evaluate the correlation between survival outcomes and potential prognostic factors, with hazard ratios (HRs) and 95% confidence intervals (CIs) for the multivariate analysis. A *p*-value of less than 0.05 was deemed statistically significant for all analyses. Survival analysis was carried out with the Kaplan–Meier test. There were missing data within the cells, but only available data were included for the rates.

Approval was obtained from the local ethics committee of Tepecik Education and Research Hospital (2024/02-22), ensuring compliance with the guidelines of the Declaration of Helsinki. Since the study was a retrospective case study, informed consent was not required from the patients.

## 3. Results

### 3.1. Patient Information

This study included 722 patients. Of these patients, 161 (22.3%) were HER2-null, 171 were (23.7%) ultralow, 249 (34.5%) were HER2 (+1), and 141 (19.5%) HER2 (+2) were negative by FISH. We grouped patients with HER2 scores of +1 and +2 together as a single group, calling them “HER2-low”, which amounted to 390 patients overall. The clinicopathological characteristics of the overall group and their distribution according to HER2 groups are shown in Table 2 and Table 3, respectively.

**Figure 1:**
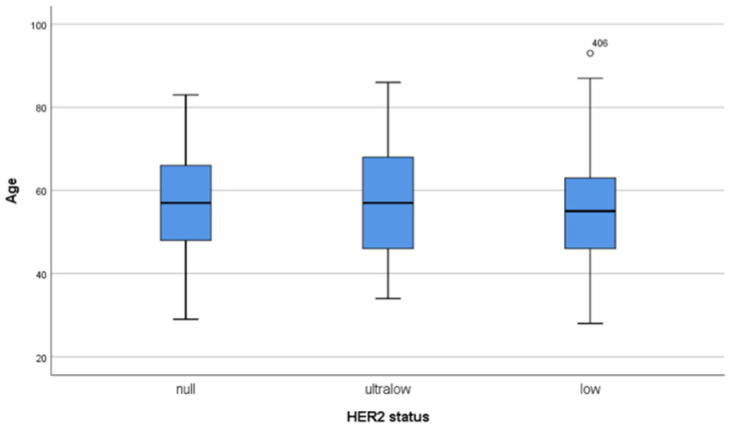
Average age (horizontal lines in the boxes) and age distribution are drawn with simple box-plots, which show the similarity between HER2-null, HER2-ultralow, and HER2-low groups.

The mean age of the overall patients was 56.16 ± 12.64 years, within a range of 28–93 years (Figure 1). The mean age did not differ among the HER2 groups. There were no significant differences in menopausal status. The clinical and pathological characteristics of the overall group and their distribution according to HER2 groups are shown in Table 2, Table 3, Table 4, Table 5, respectively.

### 3.2. Tumor Information

There were no significant differences in tumor side (left/right), tumor number (single/multiple), histological types (ductal, lobular, or others), or the nuclear or histological grade of the tumor among the groups. Metaplastic tumors exhibited HER2-low expression. Among the papillary histology cases (*n* = 14), none showed HER2-ultralow expression; seven were HER2-null, and seven were HER2-ultralow. Mucinous histology varied across HER2 groups, with two tumors being HER2-null, three being HER2-ultralow, and the remaining one being HER2-low.

**Figure 2:**
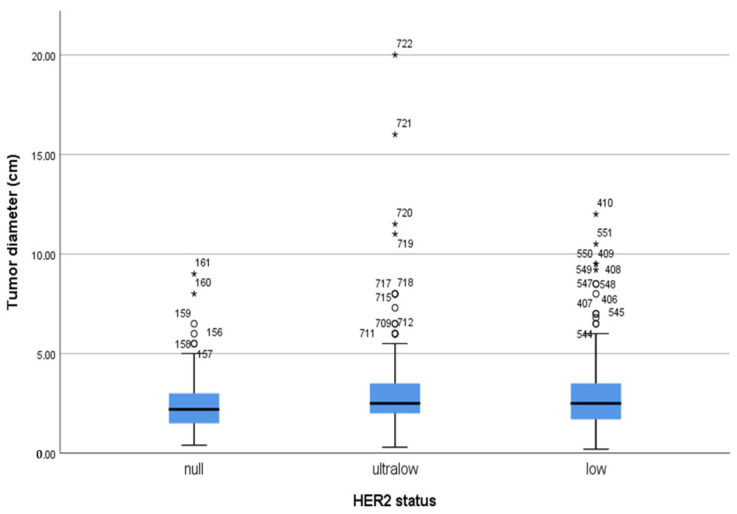
Mean values (horizontal lines in the boxes) and the distribution of the tumor diameters of patients in the HER2-null, HER2-ultralow, and HER2-low groups are shown by a simple box-plot graph with extreme values. The blue boxes represent the middele 50% of the data. Potential outliers are marked with a circle (°) and extreme outliers are marked with an asterisk (*).

The mean tumor diameter of the entire cohort was 2.85 ± 1.82 cm, with a range of 0.2–20 cm. While two-thirds of the patients were evaluated as having T2 tumors, T4 tumors constituted only 2.4% of all patients. HER2-ultralow tumors have the largest mean tumor diameter at 3.11 cm. HER2-low tumors follow with a mean diameter of 2.88 cm. In contrast, HER2-null tumors have the smallest mean tumor diameter, measuring 2.54 cm. However, the higher extreme values observed in the HER2-ultralow group may be a contributing factor to the observed differences in tumor size (Figure 2). Nevertheless, no significant differences were observed between the groups when evaluated according to T stage (*p* = 0.197).

### 3.3. Tumor Nature

There were no significant differences among the groups in terms of the presence or absence of necrosis and DCIS.

**Figure 3:**
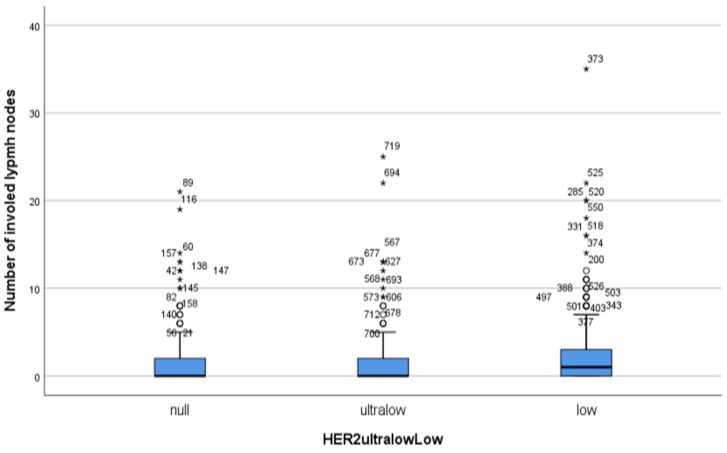
Mean number of involved lymph nodes (horizontal lines in the boxes) represented by simple box-plot graphs is shown according to HER2-null, HER2-ultralow, and HER2-low groups with extreme values. The blue boxes represent the middele 50% of the data. Potential outliers are marked with a circle (°) and extreme outliers are marked with an asterisk (*).

N0 represents the majority of cases, with HER2-low being the most prevalent expression, comprising over half of the cases. The distribution of lymph node involvement was found to be similar among the groups (*p* = 0.540). In cases with 1–3 involved LNs, HER2-low remained dominant, but the rate of HER2-ultralow expression increased slightly, suggesting a modest rise in HER2 expression levels with moderate disease progression. In cases with 4–9 involved LNs, HER2-low remained the primary expression, but there was a relative increase in the rate of HER2-ultralow and HER2-null cases compared to earlier stages. In the most advanced stage with ≥10LNs involved, HER2-null showed the highest proportion, followed by HER2-ultralow, indicating greater variability in HER2 expression levels as lymph node involvement becomes extensive. Although the HER2-ultralow group involved more LNs than the HER2-null group, the HER2-low group involved more LNs than the HER2-null and HER2-ultralow groups, numerically. However, this difference was not significant between the subgroups (*p* values 0.999, 0.932 and 0.976, respectively) (Figure 3). Likewise, no difference was observed among the groups when evaluated according to lymph node stage (*p* = 0.540).

### 3.4. Hormonal Receptor Status and Ki67

**Figure 4:**
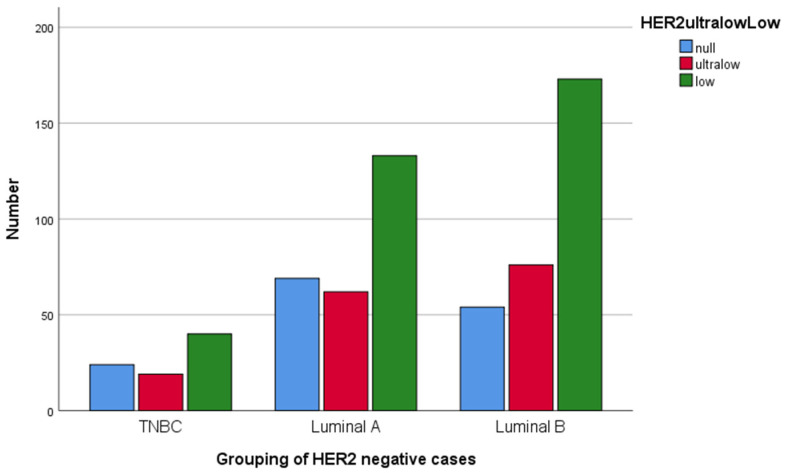
Distribution of HER2-null, HER2-ultralow, and HER2-low cases according to TNBC or Luminal A and B groups in bar charts (*p* = 0.097).

Of the overall HER2-negative group, 10.8% consisted of TNBC (*n* = 78). Of the HR-positive patients, seven patients showed ER negativity but PR positivity and three of them showed very low levels of PR expression (2%, 5%, and 5%). Among these seven patients, HER2 expression was heterogenous; one showed no expression (HER2-null), three of them showed very low expression (HER2-ultralow), and three of them were HER2-low. Basal-like rates were calculated to be slightly higher than TNBC rates when ER < 1%, but tumors with PR < 20% expressed low Ki67 levels (<14%). Hormone-positive tumors that showed missing Ki67 data were not categorized as Luminal A or B. There were no significant differences in terms of being Luminal A or B, or basal-like tumors either; these accounted for 36.6%, 42.0%, and 11.5% of the overall group, respectively, with a missing data rate of 10.0% percent (Figure 4).

There were no differences among the groups in terms of estrogen and progesterone receptor status (being positive or negative) or mean percentages of them.

**Figure 5:**
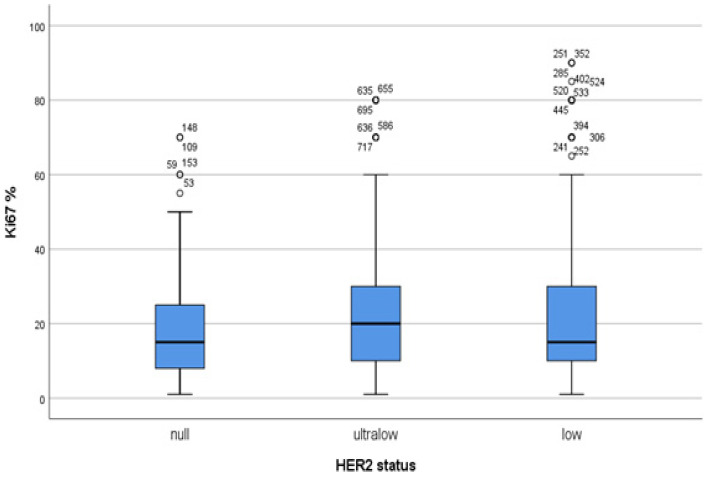
The mean Ki67 percentage (horizontal lines in the boxes) and the distribution of the Ki67 percentage represented by a simple box-plot graph are shown together with extreme values according to the HER2-null, HER2-ultralow and HER2-low groups. The blue boxes represent the middele 50% of the data. Potential outliers are marked with a circle (°).

A Kolmogorov–Smirnov test revealed that the distribution of Ki67 percentages between the HER2 groups did not exhibit a normal pattern (*p* < 0.001). A post hoc analysis of the groups revealed that both the HER2-ultralow and HER2-low groups exhibited higher Ki67 values than the HER2-null group. However, this difference did not reach statistical significance, as indicated by *p*-values of 0.052 and 0.066, respectively (Figure 5). When Ki67 percentages were grouped as low and high with a cut-off value of 20% and 30%, there were no differences between the HER2-null, HER2-ultralow, and HER-low groups (*p*-values were 0.181 and 0.095, respectively).

### 3.5. Relaps and Metastasis

**Figure 6:**
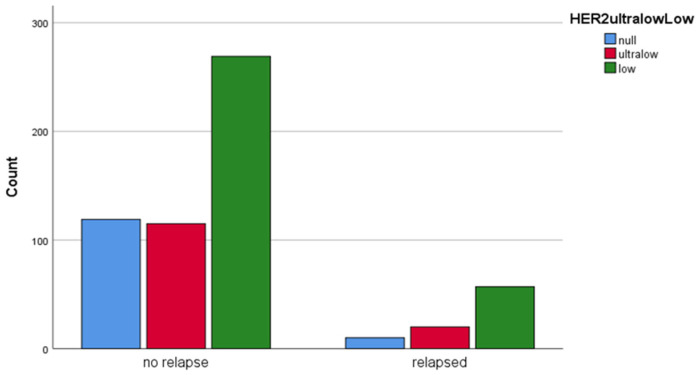
Distribution of HER2-null, HER2-ultralow, and HER2-low cases according to being relapsed or not in bar charts.

The HER2-low group had more recurrences than other groups. No statistically significant difference in recurrence rates was observed between the HER2-ultralow group and either the HER2-null group (*p* = 0.237) or the HER2-low group (*p* = 0.741). However, the HER2-low group was more likely to relapse than the HER2-null group (*p* = 0.023) (Figure 6).

Local recurrence; bone, liver, lung, or brain metastasis; survival or death at the end of at least 5 years of follow-up; and the surgical procedure performed did not differ between the groups. However, it is noteworthy that 13 of 16 patients with local recurrence, 41 of 64 patients with bone metastases, 15 of 22 patients with lung metastases, 23 of 36 patients with liver metastases, and 9 of 12 patients with brain metastases were in the HER2-low group. The low number of patients with metastases was thought to be a factor in not reaching statistical significance. However, 56% of the patients who showed relapse were in the HER2-low group, and the development of relapse was statistically significantly higher in the HER2-low group (*p* = 0.031) (Table 2).

### 3.6. Survival Rates

**Figure 7:**
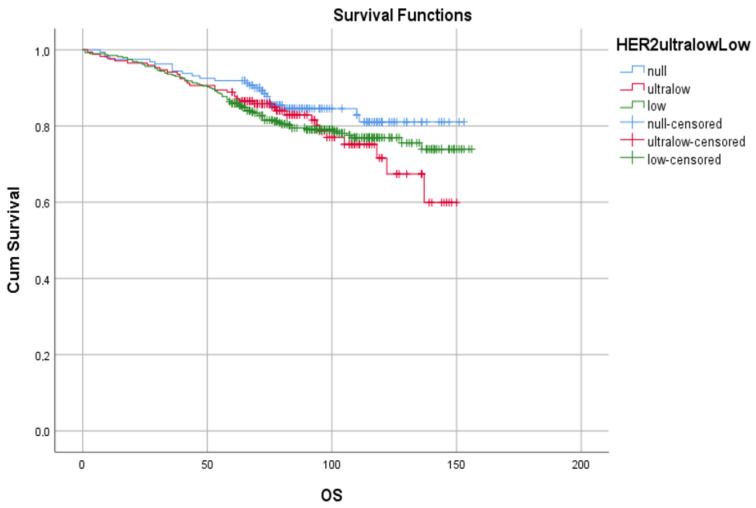

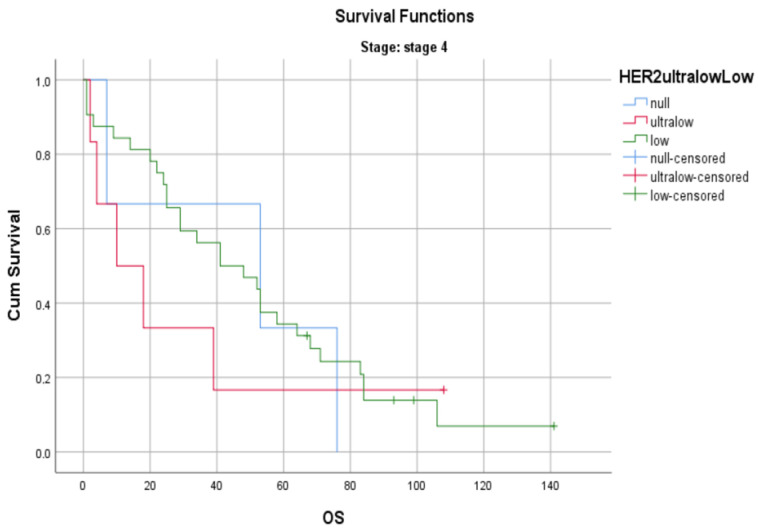
Overall survival is shown in the groups of HER2-null (blue lines), HER2-ultralow (red lines), and HER2-low (green lines) patients compared to the overall population and those metastatic disease, respectively.

**Figure 8:**
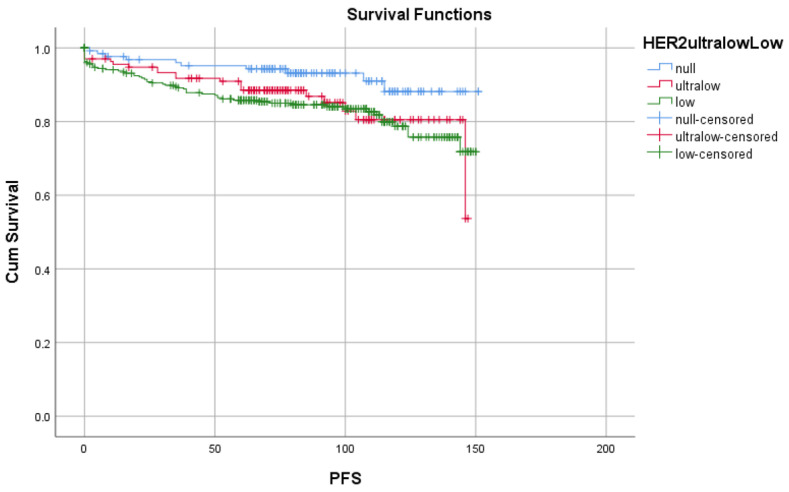
Progression-free survival (PFS) is shown in the groups of HER2-null (blue lines), HER2-ultralow (red lines), and HER2-low (green lines) patients compared to overall population (overall: 131.6 months [127.8–135.3 months]; CI:95%; log rank *p* = 0.43).

The OS rates were similar among the groups and in the overall population (overall: 132.6 months [129.1–136.1 months]; CI:95%; log rank *p* = 0.317) and in metastatic patients (overall: 48.6 months [36.0–61.2 months]; CI:95%; log rank *p* = 0.731) (Figure 7). But in terms of progression-free survival rates, HER2-null patients showed better results (overall: 131.6 months [127.8–135.3 months]; CI:95%; log rank *p* = 0.43) (Figure 8). The progression rates in metastatic patients were too small to analyze (HER2-null: 0; HER2-ultralow: 4 patients; HER2-low: 23 patients).

## 4. Discussion

The definition of “HER2-low” breast cancer has opened a new chapter in the methodology of breast cancer. Given that patients with HER2-low breast cancer showed efficacy with T-Dx, these results are encouraging for investigating the potential of this group of patients, so-called “HER2-negative”, and reclassifying them. However, data about HER2-null breast cancer are still limited. Ultimately, the similarities and differences between the HER2-null, HER2-ultralow, and HER2-low groups within the HER2-negative breast cancer cases remain to be elucidated.

Since HER2 status can be affected by cancer treatment, especially anti-HER2 therapies, those who had received breast cancer or other cancer treatment within 5 years before diagnosis were not included in this study. It has been demonstrated that chemotherapy, radiotherapy, and endocrine therapy may upregulate the expression of HER2 in HER2-negative or HER2-low breast cancer tumors through epigenetic mechanisms or the activation of adaptive mechanisms within the NF-κB pathway, which emphasizes the necessity for more rigorous assessment of HER2-negative and HER2-low tumors for subsequent lines of therapeutic options [6,7,8].

While preclinical studies have shown that adjuvant trastuzumab in the absence of HER2 amplification drives supraphysiological HER2-expressing luminal breast cancer stem cells, this has failed in clinical trials [9]. Adding trastuzumab to adjuvant chemotherapy did not improve invasive disease-free survival or OS in HER2-negative breast cancer in clinical trials, which showed 1+ or 2+ staining on immunohistochemistry for HER2, which is FISH-negative [10]. Pertuzumab monotherapy also showed no efficacy in treating HER2-negative breast cancer. As a matter of fact, the use of HER2-targeted therapies was limited to HER2-positive tumors [11].

Despite the current limitations of conventional anti-HER2 therapies in the treatment of HER2-positive breast cancer patients, the efficacy of new-generation HER2-targeted drug–antibody conjugates has been demonstrated in tumors that express HER2, albeit at low levels. The favorable outcomes of preclinical studies on HER2-targeted drug–antibody conjugates, such as trastuzumab duocarmycin and trastuzumab deruxtecan, in HER2-expressing tumors in the absence of HER2 amplification have generated considerable enthusiasm in the context of clinical trials [12,13,14].

The DESTINY-Breast04 study yielded promising outcomes with 9.9 vs. 5.1 months in terms of PFS and 23.4 vs. 16.8 months in terms of OS in patients with pretreated HER2-low metastatic breast cancer who were treated with deruxtecan compared to the physician’s choice of chemotherapy [15]. However, the desired response was not obtained with TDM1 in HER2-low breast cancer, which is also a drug–antibody conjugate [16]. Deruxtecan can cross the cell membrane independently of HER2 expression and induce apoptosis in neighboring tumor cells. This phenomenon, called the by-stander effect, explains the activity of deruxtecan in HER2-low tumors compared to previous HER2-targeting agents, including T-DM1 [17]. The ongoing DESTINY-Breast06 study is expected to define the treatment efficacy of ADCs in the HER2-ultralow group [18].

There are limited data on HER2-ultralow breast cancer in the literature. In our study, we found that survival, recurrence rates, and clinicopathological features were similar among the HER2-null, HER2-ultralow, and HER2-low groups, whereas the HER2-null group showed better survival and the HER2-low group showed worse outcomes in recurrence rates. These findings confirm the results of the only previous study by Guan et al. [19]. Additionally, we observed a higher rate of lymph node metastasis in the HER2-ultralow group, as well as a larger tumor size compared to both the HER2-null and HER2-low groups. This study expands on current knowledge on the characteristics of the HER2-ultralow group. There are no specific suggestions for the management of the HER2-ultralow group. The answer to whether the TDx response observed in the HER2-low group will also extend to the HER2-ultralow group will be provided by the results of the DAISY study, which investigates TDx response independently of HER2 status, and the DESTINY-Breast06 study, which examines responses in tumors with low HER2 expression [20].

Studies about HER-null and HER2-low breast cancer widely differ; some do not show survival differences, and some have favorable outcomes with HER2-low disease [21,22]. A good example of this was a Chinese study with 1363 cases, which found no prognostic expression in terms of OS and PFS differences among these groups [23]. However, the observation that recurrence rates are higher in the HER2-low group can be regarded as having a detrimental impact on prognosis. HER2-low breast cancer shows lower pCR rates than HER2-null tumors after neoadjuvant chemotherapy [22]. On the other hand, the administration of chemotherapy (either eribulin or capecitabine) does not result in any OS or PFS benefit for pretreated HER2-null and HER2-low patients, but the objective response rates were consistently lower in the HER2-low group [24]. Among HER2-low tumors, luminal B tumors show more aggressive pathological results, such as higher Ki67 levels, than Luminal A tumors, which translate into better results from chemotherapy, especially higher pathological response rates to neoadjuvant chemotherapy [25]. It is worth noting that HER2-low tumors mostly tend to be hormone-positive [26].

HER2 expression is maintained at low levels in normal breast cells for cell growth and differentiation, whereas overexpression acts as a driver in breast cancer [27]. The fact that low levels of expression lead to differentiation similar to normal breast cells indicates that HR is the dominant driver in luminal tumors, independent of these HER2 levels. However, in tumors without HR or HER2 expression, tumors without such a driver tend to be more aggressive [26]. Nevertheless, when such patients’ management is driven predominantly by chemotherapy, this may translate into better survival functions, as shown in our study. But other studies resulted in inferior results in HER2-null breast cancer survival rates compared to HER2-low breast cancer survival rates [28].

Triple-negative breast cancer, characterized by the absence of expression of ER, PR, and HER2, accounts for 10–15% of all breast cancer cases. Currently, standardized protocols for assessing HER2-null, HER2-ultralow, and Her2-low expression in breast cancer are lacking. Traditionally, classification has been binary, categorizing tumors as either HER2-positive or HER2-negative, without consideration of intermediate expression levels. However, when coming across the term “HER2-null” in the literature, we may rename the TNBC group as “*true TNBC*”. On the other hand, advancements in treatment approaches in HER2-low tumors may lead to the classification of HER2-low tumors as a distinct subgroup within HER2-expressing tumors, similarly to the subdivision of HR-positive tumors into luminal groups.

Evaluating the differences between the HER2-low, HER2-ultralow, and HER2-null group solely based on biological markers may not be sufficient. Emerging evidence suggests that micronutrient levels play a significant role in breast cancer progression [29]. Investigating the metabolic profiles specific to HER2 subtypes could provide deeper insights into tumor biology and contribute to the development of personalized therapeutic approaches.

It may become imperative to report the actual level of expression when ADC compounds become an option in the treatment of HER2-expressing tumors. However, there is a growing consensus that the HER2-negative group should be reconsidered and that new techniques or nomenclature for classification may be required [6,22,30,31]. Nevertheless, according to our data, we can conclude that although HER2-low tumors are differentiated in terms of treatment, this does not result in differences in clinicopathological features and has little effect on survival.

This study has some limitations. Specimens may have been obtained from the primary tumor or from metastases. This may be an issue in the context of HER2 heterogeneity. Specimens were evaluated by the same pathologist. These single-center data were collected retrospectively, so some of the analyses were conducted by excluding missing data.

## 5. Conclusions

Significant advances have been made in the management of breast cancer to date. As our understanding of tumor pathogenesis and molecular targets deepens, personalized cancer therapy is becoming the norm, superseding empirical treatments. This shift under-scores the necessity to more accurately delineate the biological underpinnings of cancer. It is crucial to gain insight into the characteristics of and similarities and differences in each subgroup in order to more accurately identify and recognize HER2-negative cases.

The high number of relapses in the HER2-low group compared to the HER2-null and HER2-ultralow groups is an indication that new treatment modalities are needed in this group. The results from our novel study aim to help address relevant unmet needs for clinical data in HER2-negative breast cancer patients in terms of the HER2-null, HER2-ultralow, and HER2-low subgroups and may lead to new therapeutic approaches or the development of new diagnostics in this field.

## Figures and Tables

**Table 1:** Definition of HER2-null, HER2-ultralow, and HER2-low breast cancer tumors.

	Result	Definition
HER2-null	Negative (Score 0)	Complete absence of staining of tumor cells
HER2-ultralow	HER2-ultralow	Incomplete membrane staining that is faint/barely perceptible and within ≤10% of tumor cells
HER2-low	Negative (Score 1+)	Incomplete membrane staining that is faint/barely perceptible and within >10% of tumor cells
	Equivocal (Score 2+) and HER2 ISH negative	Weak to moderate complete membrane staining in >10% of tumor cells or complete membrane staining that is intense but within ≤10% of tumor cells

**Table 2:** Clinical characteristics of our patients. BCT: breast-conserving therapy; ALND: axillary lymph node dissection; SLND: sentinel lymph node dissection; HER2: human epithelial receptor 2; mastectomy includes modified radical mastectomy and simple mastectomy; TNBC: triple-negative breast cancer; ER: estrogen receptor; PR: progesterone receptor; DCIS: ductal carcinoma in situ.

		Frequency	Percent
Relapse	No	503	69.7
	Yes	87	12.0
	Missing	132	18.3
Local recurrence	No	572	79.2
	Yes	16	2.2
	Missing	134	18.6
Bone metastasis	No	528	73.1
	Yes	64	8.9
	Missing	130	18.0
Pulmonary metastasis	No	569	78.8
	Yes	22	3.0
	Missing	131	18.1
Liver metastasis	No	556	77.0
	Yes	36	5.0
	Missing	130	18.0
Cranial metastasis	0	578	80.1
	1	12	1.7
	Missing	132	18.3
Menopause status	Pre-	277	38.4
	Post-	412	57.1
	Missing	33	4.6
Sidedness	Right side	316	45.9
	Left side	373	54.1
	Missing	33	4.6
Tumor number	Single	513	71.1
	Multiple	102	14.1
	Missing	107	14.8
Operation type	BCT + SLND	510	70.6
	BCT + ALND	24	3.3
	Mastectomy	188	26.0
Treatment	Endocrine therapy	125	17.3
	Endocrine therapy + Chemotherapy	396	54.8
	Chemotherapy	44	6.1
	Missing	156	21.8
Survey	Ex	143	19.8
	Alive	579	80.2

**Table 3:** Pathological characteristics of our patients. Others: * histological subtype refers to metaplastic (*n* = 1), mucinous (*n* = 14), and papillary (*n* = 5) histology. HER2: human epithelial receptor 2, mastectomy includes modified radical mastectomy and simple mastectomy; TNBC: triple-negative breast cancer; ER: estrogen receptor; PR: progesterone receptor; DCIS: ductal carcinoma in situ.

		Frequency	Percent
Hormone status	TNBC	78	10.8
	Hormone positive	644	89.2
Luminal/ nonluminal	Basal like	83	11.5
	Luminal A	264	36.6
	Luminal B	303	42.0
	Missing	72	10.0
HER2 status	Null	161	22.3
	Ultralow	171	23.7
	1+	249	34.5
	2+	141	19.5
ER status	Negative	85	11.8
	1+	24	3.3
	2+	126	17.5
	3+	487	67.5
PR status	Negative	119	16.4
	1+	44	6.1
	2+	134	18.6
	3+	425	58.9
	Missing	93	12.9
DCIS	Absent	220	30.5
	Present	502	69.5
Necrosis	Absent	592	82.0
	Present	130	18.0
Involved lymph nodes	0	350	48.5
	1–3	198	27.4
	4–9	92	12.7
	>10	33	4.6
Histological grade	1	39	5.4
	2	467	64.7
	3	216	29.9
Nuclear grade	1	45	6.2
	2	466	64.5
	3	211	29.2
Histological subtype	Ductal	649	89.9
	Lobulary	53	7.3
	Others *	20	2.8

**Table 4:** Clinical characteristics are shown according to HER2-null, HER2-ultralow, and HER2-low status with significant values. *p* values under 0.05 are bolded.

	HER2-Null	HER2-Ultralow	HER2-Low	*p*-Value
Age (years)				
Mean	57.17	57.32	55.24	0.104
Range	29–83	34–86	28–93	
Menopause status (*n*)				
Premenopausal	54	69	154	0.336
Postmenopausal	100	96	216	
Tumor site				
Right	61	85	170	0.127
Left	92	81	200	
Tumor number				
Single	126	121	266	0.101
Multiple	22	16	64	
Tumor stage				
T1	57	41	109	0.197
T2	90	108	233	
T3	12	15	40	
T4	2	7	8	
Tumor diameter (cm)				
Mean	2.54	3.11	2.88	**0.016**
Std. deviation	1.41	2.37	1.67	
Involved lymph nodes				
0	87	83	180	0.54
1–3	38	44	116	
4–9	18	21	53	
10 or more	10	8	15	
Stage				
Stage 1	47	36	93	**0.045**
Stage 2	77	85	181	
Stage 3	26	32	65	
Stage 4	3	6	32	
Local recurrence				
No	128	132	312	0.073
Yes	1	2	13	
Metastasis status				
Absent	150	153	339	**0.006**
Present	3	6	32	
Relapse				
No	119	115	269	**0.031**
Yes	10	20	57	
Surgical procedure				
BCT + SLND	120	123	267	0.686
BCT + ALND	5	5	14	
MRM	25	34	67	
SM	11	9	42	
Treatment				
Endocrine therapy	27	33	65	0.289
Chemo + endocrine therapy	81	87	228	
Chemotherapy	11	9	24	
Watchful waiting	1	-	-	
Brain metastasis				
No	129	132	317	0.3
Yes	2	1	9	
Bone metastasis				
No	123	119	286	0.133
Yes	8	15	41	
Pulmonary metastasis				
No	130	128	311	0.062
Yes	1	6	15	
Liver metastasis				
No	128	125	303	0.119
Yes	3	10	23	
Survey				
Exitus	25	35	83	0.296
Surviving	136	136	307	

**Table 5:** Pathological characteristics of our patient, shown according to HER2-null, HER2-ultralow, and HER2-low status with significant values.

Tumor cell type				
Ductal	141	152	356	0.3
Lobular	12	16	25	
Others *	8	3	9	
Tumor nuclear grade				
Grade 1	10	10	25	0.991
Grade 2	103	109	254	
Grade 3	48	52	111	
Histological grade				
Grade 1	7	7	25	0.637
Grade 2	108	115	244	
Grade 3	46	49	121	
Necrosis				
Absent	133	135	324	0.49
Present	28	36	66	
DCIS				
Absent	55	53	112	0.444
Present	106	118	278	
ER positivity				
Negative	25	19	41	0.24
Positive	136	152	349	
ER %				
Mean	82. 63	84.55	83.91	0.069
Std deviation	21.56	17.2	20.11	
PR positivity				
Negative	35	27	57	0.118
Positive	126	144	333	
PR %				
Mean	71.86	70.68	6.56	0.079
Std deviation	29.96	27.88	32.51	
HER2 negative				
Triple negative	24	16	38	0.162
Hormone positive	137	155	352	
Luminal types				
Basal like	24	19	40	0.097
Luminal A	69	62	133	
Luminal B	54	76	173	
Ki67 %				
Mean	18.21	22.95	22.11	0.086
Std deviation	14.86	18.35	18.38	
Ki67 (Low/High)				
<20%	87	78	182	0.181
<30%	114	106	251	0.095

Others: * histological subtype refers to metaplastic (*n* = 1), mucinous (*n* = 14), and papillary (*n* = 5) histology.

## Data Availability

Due to ethical restriction rules of the instution and privacy, data-sharing is not necessary.

## Abbreviations

The following abbreviations are used in this manuscript:

TNBC	Triple-negative breast cancer
HER2	Human epithelial receptor 2
ER	Estrogen receptor
PR	Progesterone receptor
IHC	Immunohistochemistry
ISH	In situ hybridization
DCIS	Ductal carcinoma in situ
HR	Hazard ratio
LNs	Lymph nodes
ALND	Axillary lymph node dissection
SLND	Sentinel lymph node dissection
BCT	Breast conserving therapy
OS	Overall survival
